# The Recent Typhoid Epidemic in Paris

**Published:** 1883-01

**Authors:** 


The Recent Typhoid Epidemic in Paris.—Every visitation of typhoid fever in the form of an epidemic is marked by its own peculiar characteristics. While the disease runs its cyclical course with a regular appearance of the eruption, a typical range of temperature and characteristic diarrhœal discharges, the differences between one epidemic and another will be found in the complications. The peculiarity of the epidemic which has just come to an end in Paris did not consist in a very low mortality rate, although the disease produced no such ravages as former visitations.

The complications that were most marked were thrush, bed-sores and febrile recrudescence during convalescence. If the question were asked: had therapeutics a marked influence in the production of such favorable results in this dreaded epidemic, the answer would be decidedly and unreservedly in the negative. The number of deaths, Bourneville, the editor of the *Progres Medical*, states has doubled since 1871. Therefore if therapeutics has made advances, death has made still greater.

The Parisian physicians are not as enthusiastic over quinine, boric and phenic acids and salicylate of soda as they were a few months ago. In fact, they question their efficiency. The results of their treatment, which they call "armed expectation"—another term for the "expectant plan"—are shown, they affirm, in the few complications of the fever. It is well known that the prognostic data in typhoid depend in great measure upon the presence or absence of complications. The fever runs a course that cannot be shortened; you may guide it, but like a runaway horse it must stop of itself. Hence to sit by and carefully watch, to be on the lookout for complications, is the chief element in the treatment practiced by the French during the last few weeks.

The theory that typhoid fever is a "*vaccinnate*" malady seems to have received new impetus from certain developments in the epidemic of which we are speaking. As additional proof of its being vaccinante they adduce the fact of its rarely attacking an individual the second time. On this side of the water not much stress is laid upon this, which is *not,* besides, a phenomenal occurrence, as a glance at any of the numerous medical text-books will show.

With characteristic rapidity the French *savants* seem to have deduced a number of theories regarding, and to have advocated distinct changes in the treatment of, typhoid fever, a disease peculiarly and primarily associated with pathology in France.

